# Nitrogen-Doped Carbon Dot/TiO_2_ Hybrid Composites Induce Light-Dependent ROS-Mediated Cytotoxicity in Cancer Cells

**DOI:** 10.3390/biom16091229

**Published:** 2026-08-24

**Authors:** Assia Azouaghe, Florence Back, Walid Daoudi, Abdelmalik El Aatiaoui, Céline Spack, Diana Potes Vecini, David Hoogewijs

**Affiliations:** 1 Laboratory of Molecular Chemistry, Materials and Environment (LCM2E), Department of Chemistry, Faculty of Applied Sciences, University Mohamed I, Nador 60700, Morocco; assia.azouaghe@ump.ac.ma (A.A.); a.elaatiaoui@ump.ac.ma (A.E.A.); 2 Section of Medicine, Department of Endocrinology, Metabolism and Cardiovascular System, University of Fribourg, Chemin Du Musée 5, 1700 Fribourg, Switzerland; celine.spack@unifr.ch; 3 Laboratory of Education, Sciences and Techniques, Higher School of Education and Training of Berrechid, Hassan First University, Settat 26100, Morocco; walid.daoudi@ump.ac.ma; 4 Department of Chemistry, University of Fribourg, Chemin Du Musée 9, 1700 Fribourg, Switzerland; diana.potesvecini@unifr.ch; 5 Swiss National Center for Competence in Research (NCCR) Bio-Inspired Materials, University of Fribourg, Chemin des Verdiers 4, 1700 Fribourg, Switzerland

**Keywords:** nitrogen-doped carbon dots (N-CDs), composites, photodynamic therapy (PDT), reactive oxygen species (ROS), A549 lung cancer cells, Kelly neuroblastoma cells

## Abstract

Photodynamic therapy (PDT) exploits photoactivated materials that generate reactive oxygen species (ROS) to induce selective cancer cell death. Nitrogen-doped carbon dots (N-CDs) have emerged as promising photosensitizers owing to their favorable optical properties, while hybridization with titanium dioxide (TiO_2_) may further enhance photoinduced ROS generation through improved charge separation. Here, we synthesized a series of N-CD/TiO_2_ hybrid composites with varying TiO_2_ content using a hydrothermal approach and systematically investigated the relationship between their physicochemical characteristics and biological activity. The hybrid materials were characterized by Fourier-transform infrared spectroscopy, X-ray diffraction, scanning electron microscopy, dynamic light scattering, and UV–visible spectroscopy. Among the formulations investigated, the composite containing 90% N-CDs and 10% TiO_2_ (N-CDs10T) exhibited the smallest hydrodynamic diameter, a relatively narrow particle size distribution, favorable optical properties, and the strongest irradiation-dependent biological responses. Biological activity was evaluated in A549 lung adenocarcinoma and Kelly neuroblastoma cells. Under dark conditions, all formulations displayed relatively low intrinsic cytotoxicity. Following irradiation with 365 nm UVA light, however, N-CDs10T induced a marked increase in intracellular ROS production, activation of antioxidant response element (ARE)-dependent signaling, disruption of cell-cycle progression, apoptosis-associated cell death, and inhibition of cell proliferation and migration. Kelly cells exhibited greater sensitivity than A549 cells, with IC_50_ values decreasing from 0.98 mg/mL under dark conditions to 0.52 mg/mL following irradiation. Collectively, these findings demonstrate that N-CD/TiO_2_ hybrid composites function as photoresponsive materials that enhance ROS-mediated cytotoxicity upon light activation. Beyond demonstrating phototoxicity, this study systematically links hybrid composition with oxidative stress signaling and multiple cellular responses, providing a comprehensive biological evaluation of N-CD/TiO_2_ hybrid materials. While additional studies are required to identify the predominant ROS, evaluate selectivity in non-malignant cells, and optimize activation at clinically relevant wavelengths, the present work establishes a proof of concept for the development of N-CD/TiO_2_ hybrid composites for photodynamic applications.

## 1. Introduction

Carbon dots (CDs) have emerged as a versatile class of carbon-based nanomaterials owing to their unique optical properties, excellent aqueous dispersibility, favorable biocompatibility [1], and straightforward synthesis from inexpensive precursors. Since their discovery in 2004 [2], CDs have attracted considerable interest for applications in bioimaging, biosensing, catalysis, drug delivery, and regenerative medicine [3]. More recently, their ability to generate reactive oxygen species (ROS) following photoactivation has positioned CDs as promising photosensitizers for photodynamic therapy (PDT) [4,5,6].

Nitrogen doping represents one of the most effective approaches to tailor the physicochemical properties of CDs [7,8]. Incorporation of nitrogen-containing functional groups modifies the electronic structure by introducing defect-associated energy states, thereby improving charge separation, fluorescence efficiency, and photoinduced electron transfer. Consequently, nitrogen-doped carbon dots (N-CDs) often exhibit enhanced ROS generation while maintaining favorable colloidal stability and low intrinsic toxicity [9], making them particularly attractive candidates for photodynamic applications [5,10].

Photodynamic therapy is based on the activation of a photosensitizer by light of an appropriate wavelength, leading to the generation of cytotoxic ROS that selectively damage malignant cells while minimizing systemic toxicity [11]. Following photoexcitation, photosensitizers may undergo intersystem crossing to a long-lived triplet state, enabling either electron transfer reactions (Type I photochemistry) or energy transfer to molecular oxygen, resulting in singlet oxygen production (Type II photochemistry). The relative contribution of these pathways depends on both the physicochemical properties of the photosensitizer and the surrounding biological environment. Consequently, considerable efforts have focused on developing novel photoresponsive nanomaterials capable of improving ROS generation and photodynamic efficacy [4,5,6].

Titanium dioxide (TiO_2_), particularly in its anatase crystalline phase, is a well-established semiconductor photocatalyst capable of generating electron–hole pairs upon ultraviolet irradiation, resulting in the formation of hydroxyl radicals, superoxide anions, and other reactive oxygen species. Although TiO_2_ possesses excellent photochemical stability and high ROS-generating capacity, rapid recombination of photogenerated charge carriers and its dependence on ultraviolet activation limit its efficiency when used alone in biomedical applications [12,13]. Hybridization with carbon-based nanomaterials has therefore emerged as an attractive strategy to improve charge separation, suppress electron–hole recombination, and enhance photocatalytic activity [6].

Among carbon-based materials, N-CDs are particularly promising partners for TiO_2_ because their abundant surface amino groups facilitate intimate electronic coupling with semiconductor surfaces while simultaneously providing tunable optical properties and efficient photoinduced electron transfer. Recent studies have demonstrated that carbon dot/TiO_2_ hybrid materials exhibit improved photochemical activity compared with the individual components, highlighting their potential for photodynamic applications [5,6]. However, relatively few studies have systematically correlated hybrid composition with both physicochemical characteristics and downstream biological responses, including oxidative stress signaling, cell-cycle regulation, apoptosis, proliferation, and migration. A better understanding of these relationships is essential for the rational design of more effective photoresponsive nanomaterials.

In the present study, we systematically investigated how the composition of nitrogen-doped carbon dot (N-CD)/TiO_2_ hybrid composites influences their physicochemical properties and photodynamic biological activity [14,15]. To this end, we synthesized a series of hybrid materials containing different TiO_2_ ratios using a simple hydrothermal approach. Following comprehensive physicochemical characterization by Fourier-transform infrared spectroscopy (FTIR), X-ray diffraction (XRD), scanning electron microscopy (SEM), dynamic light scattering (DLS), and UV–visible spectroscopy, we evaluated their biological activity in A549 lung adenocarcinoma and Kelly neuroblastoma cells. Using complementary in vitro assays, we assessed irradiation-dependent ROS generation, antioxidant response element (ARE)-mediated signaling, cell proliferation, migration, apoptosis, and cell-cycle progression. By integrating comprehensive physicochemical characterization with multiple complementary biological assays, this study systematically correlates hybrid composition with photodynamic biological responses rather than simply demonstrating phototoxicity, thereby providing a framework for the rational optimization of N-CD/TiO_2_ hybrid materials for future photodynamic applications.

## 2. Materials and Methods

### 2.1. Material

Citric acid, 2,6-diaminopyridine, titanium(IV) butoxide (Ti(OBu)_4_), nitric acid (HNO_3_), ethanol, dimethyl sulfoxide (DMSO), phosphate-buffered saline (PBS), Dulbecco’s Modified Eagle Medium (DMEM, high glucose), fetal bovine serum (FBS), penicillin–streptomycin solution, resazurin sodium salt, RNase A and all other analytical-grade chemicals were purchased from Merck (Darmstadt, Germany) unless stated otherwise. JetOPTIMUS^®^ DNA transfection reagent and JetOPTIMUS^®^ buffer were obtained from Polyplus (Illkirch, France). Passive Lysis Buffer and the Dual-Glo^®^ Luciferase Assay System were purchased from Promega (Madison, WI, USA). Annexin V-CF488A and propidium iodide were purchased from Biotium (Fremont, CA, USA). CellROX™ Green reagent and H_2_DCF-DA were used for ROS detection according to the manufacturers’ instructions. Ultrapure water (18.2 MΩ.cm) was used throughout all syntheses and analyses.

### 2.2. Methods

#### 2.2.1. Synthesis

**N-CD synthesis:** One gram of citric acid (5.20 mmol) was dissolved in 15 mL of ultrapure water. Subsequently, 1 g of 2,6-diaminopyridine (9.16 mmol) was added under magnetic stirring. After homogenization by ultrasonication, the solution was transferred to a Teflon-lined stainless steel autoclave and heated at 160 °C for 24 h. After cooling to room temperature, the reaction mixture was centrifuged (10,000 rpm, 10 min), filtered, purified by dialysis (MWCO 3500 Da), dried at 70 °C for 24 h, and ground into a fine powder to obtain nitrogen-doped carbon dots (N-CDs).

**TiO_2_ synthesis:** Titanium dioxide nanoparticles were synthesized hydrothermally. Titanium (IV) butoxide (2 g, 5.88 mmol) was dissolved in 35 mL ethanol before addition of 3.5 mL of 10% nitric acid under continuous stirring until a transparent solution was obtained. The precursor solution was transferred into a Teflon-lined autoclave and heated at 180 °C for 24 h. The resulting precipitate was collected, dried at room temperature and gently ground into a fine powder.

**N-CDs-TiO_2_ synthesis:** Hybrid N-CD/TiO_2_ composites containing different TiO_2_ weight fractions were prepared by dispersing 0.95 g, 0.90 g or 0.85 g N-CDs in 3 mL ethanol, followed by addition of 0.05 g, 0.10 g or 0.15 g TiO_2_ nanoparticles, respectively. The suspensions were sonicated and stirred overnight to promote homogeneous mixing. Following solvent evaporation, the resulting hybrid materials were designated N-CDs5T, N-CDs10T and N-CDs15T according to their TiO_2_ content.

#### 2.2.2. Characterization

**Fourier-Transform Infrared Spectroscopy:** FTIR spectra were recorded using a Shimadzu IRAffinity-1S spectrometer (Shimadzu, Kyoto, Japan) in ATR mode over the range 400–4000 cm^−1^ using a spectral resolution of 4 cm^−1^ and 32 accumulated scans.

**X-Ray Diffraction:** X-ray diffraction patterns were acquired on a Shimadzu XRD-6000 diffractometer using Cu Kα radiation (λ = 1.5406 Å) over a 2θ range of 2–80°. Diffraction peaks were assigned using the ICDD database.

**UV-Vis spectroscopy:** UV–visible absorption spectra were recorded using a Jasco V730 spectrophotometer (JASCO Corporation, Tokyo, Japan) using quartz cuvettes (1 cm path length).

**SEM:** Scanning electron microscopy was performed using a Thermo Fisher XL30S FEG microscope (Thermo Fisher Scientific, Waltham, MA, USA) operated at an accelerating voltage of 7.5 kV. Samples were sputter-coated with gold prior to imaging.

**DLS:** Dynamic light scattering measurements were performed at 25 °C using a NanoLab 3D instrument (LS Instruments, Fribourg, Switzerland). Samples were dispersed at 1 mg/mL in ultrapure water following ultrasonication and filtration through a 5 μm filter. At least nine independent measurements were performed for each sample. Refractive index = 0.89.

#### 2.2.3. Biological Activity

**Cell lines:** Human lung adenocarcinoma A549 cells (ATCC CCL-185™, Manassas, VA, USA) and Kelly neuroblastoma cells (ECACC 92110411; Merck, Darmstadt, Germany) were cultured in high-glucose Dulbecco’s Modified Eagle Medium supplemented with 10% fetal bovine serum and 1% penicillin–streptomycin at 37 °C in a humidified atmosphere containing 5% CO_2_. Cell lines were routinely tested for mycoplasma contamination and authenticated according to supplier recommendations. Different initial seeding densities were used depending on the biological assay to ensure optimal assay performance and adequate signal-to-noise ratios and to avoid overconfluence during the respective incubation periods. Consequently, cell densities were optimized according to assay duration and analytical endpoint rather than being identical across all experiments.

**Metabolic activity:** For metabolic activity measurements, cells were seeded at relatively low density to maintain logarithmic growth throughout the 24 h treatment period and to avoid confluence-related alterations in metabolic activity. A549 cells were seeded at 4 × 10^4^ cells/mL, whereas Kelly cells were seeded at 2 × 10^5^ cells/mL to account for differences in growth characteristics between the two cell lines. A 200 µL volume of 0.05 3 mg/mL solutions of particles was added and left for 24 h in a CO_2_ incubator (37 °C; 5% CO_2_). The media were removed and washed with 100 µL of PBS. A 100 µL volume of medium containing 11 µg/mL resazurin was added to each well. Plates were then read using a Biotek^®^ SynergyH1 UV-Vis spectrometer (BioTek Instruments, Inc., Winooski, VT, USA) after 1.5 h of incubation, with excitation at 554 nm and emission at 593 nm.

**Live-cell holographic imaging:** For long-term holographic imaging, a higher initial cell density was used to ensure reliable automated cell tracking throughout the 48 h recording period while avoiding excessive confluence at the end of the experiment.

**Confluence, distance and speed:** A 100,000/mL quantity of A549 cells was seeded on 6-well plates and left for 48 h in a CO_2_ incubator (37 °C; 5% CO_2_). The different concentrations of N-CD_S_15T were added to the wells, and the plate was placed in a HoloMonitor M4 holographic microscope (Phase Holographic Imaging AB, Lund, Sweden) within a CO_2_ incubator (37 °C; 5% CO_2_) in order to monitor cell confluence, distance, and speed in real time. The HoloMonitor software’s Kinetic Cell Proliferation Assay and Kinetic Cell Motility Assay modules (version 4.xx; Phase Holographic Imaging, Lund, Sweden) were used to quantify cell growth and calculate average migration speed based on the captured images.

**Detection of ROS and viability:** A 100,000/mL quantity of A549 cells (P51) was seeded on 96-well plates and left for 48 h in a CO_2_ incubator. Different concentrations of N-CDs10T were added, and the cells were incubated for another 24 h. The media were removed and washed with 100 µL of PBS. A negative control was performed with cells without N-CDs and without irradiation, a positive control with cells and 0.5% H_2_O_2_ without irradiation, a blank control with N-CDs10T without irradiation, and a probe control with irradiation without N-CDs10T. A concentration of 2.5 μM CellROX^TM^ or 10 μM H_2_DCF-DA was added and left to incubate for 1 h. The media were removed and washed with 100 µL of PBS. A 100 μL volume of PBS was added with a new concentration of 2.5 μM CellROX^TM^ or 10 μM H_2_DCF-DA in PBS. The cells were irradiated for 80 min and 20 min (Kelly) with the Alonfire^®^ H42 UV lamp, Shenzhen Shiwang Technology Co., Ltd., Shenzhen, China (6 W, 365 nm, 5 cm, 0.56–0.93 (J/cm^2^)/min (estimated)). Plates were then read using a Biotek^®^ SynergyH1 UV-Vis spectrometer with excitation at 490 nm and emission at 520 nm. The media were removed and washed with 100 µL of PBS. A 100 µL volume of medium containing 11 µg/mL resazurin was added to each well. Plates were subsequently read using a Biotek^®^ SynergyH1 UV-Vis spectrometer after 1.5 h of incubation, with excitation at 554 nm and emission at 593 nm, as described previously [16]. 

**Assessment of the source of ROS**: A 75,000/mL quantity of A549 (P21) cells was seeded on 96-well plates and left for 24 h in a CO_2_ incubator. A 0.77 mg/mL quantity of N-CDs10T, TiO_2_ and N-CDs was added, and the cells were incubated for another 24 h. A concentration of 2.5 μM CellROX^TM^ or 10 μM H_2_DCF-DA was added and left to incubate for 1 h. The media were removed and washed with 100 µL of PBS. A 100 μL volume of PBS was added with a new 0.77 mg/mL composite. The cells were irradiated for 20 min. A 100 μL volume of PBS was added with a new concentration of 2.5 μM CellROX^TM^ or 10 μM H_2_DCF-DA in PBS. Plates were then read using a Biotek^®^ SynergyH1 UV-Vis spectrometer with excitation at 490 nm and emission at 520 nm.

**Luciferase reporter gene assays:** A549 cells (6 wells; 10^4^ cells/mL) were transfected with ARE-driven firefly luciferase (4.34 ng/mL) and *Renilla* plasmids (0.38 ng/mL) using JetOPTIMUS^®^. After 24 h, cells were treated with 0.57 mg/mL N-CDs10T. On day 4, cells were irradiated at 365 nm for 20, 40, or 60 min to induce oxidative stress. Controls included a negative PBS group and a dark (non-irradiated) group. Cells were then lysed using Passive Lysis Buffer (Promega) and stored at −20 °C. Luciferase activities of triplicate wells were determined using the Dual Luciferase Reporter Assay System, as described before [17].

**Cytometry/cell-cycle analysis:** Flow cytometric analysis requires isolated single-cell suspensions and was therefore performed using a lower initial seeding density to minimize cell clustering and facilitate accurate cell-cycle analysis following treatment. A 20,000/mL quantity of A549 cells was seeded on 96-well plates and left for 48 h in a CO_2_ incubator (37 °C; 5% CO_2_). The cells were treated with 1.5 mg/mL of N-CD_S_10T for 24 h. The wells were washed with PBS, 500 µL of PBS was added, and 4 mL of 70% ethanol (−20 °C) was added dropwise in an ice bath. The cells were fixed for 30 min and centrifuged for 5 min at 500× *g*. The solvent was aspirated, and 500 µL of a PBS solution containing 50 µg/mL propidium iodide and 50 µg/mL RNase A was added and left to incubate at R.T. for 1 h in the dark. The tubes were used as is and read using the LSRFortessa^TM^ (BD Biosciences, San Jose, CA, USA). Data were processed using FlowJo v10.10.1 software. A total of 100,000 cells per sample were used, with triplicate samples of cells treated with N-CDs10T.

**Apoptosis/necrosis evaluation:** Cell density was selected to provide sufficient viable cells for quantitative fluorescence measurements while allowing reliable discrimination between apoptotic and necrotic populations during the time-course analysis. A 40,000/mL quantity of A549 lung cancer cells or a 200,000/mL quantity of Kelly neuroblastoma cells was seeded on 96-well plates and left for 24 h in a CO_2_ incubator (37 °C; 5% CO_2_). On the plate, one line of wells was dedicated to control cells, one line for cells treated with 1% Triton, one line for cells treated only with Annexin V, and one line for cells treated with 1.5 mg/mL of N-CD_S_10T (1 mg/mL for Kelly cells). After 24 h of incubation, the medium was changed to 1.5 mg/mL of N-CD_S_10T (1 mg/mL for Kelly cells). After the desired incubation time (3 h, 18 h, 24 h), the medium containing N-CD_S_10T was removed from the cells and transferred to adjacent wells. A 100 µL volume of HEPES buffer was added to the previously emptied and PBS-washed cell wells. In both the control wells and the N-CD_S_10T-treated wells, 10 µg/mL of PI and 0.5 µg/mL of Annexin V were added. A 10 µg/mL quantity of PI was added to the removed medium and the control well. A control medium alone plus 10 µg/mL of PI was added to the plate. Finally, 1% Triton was added to the corresponding row of wells. The plate was left in the dark for 15 min and read at 490 ex./515 em. nm for Annexin V and at 535 ex./617 em. nm for PI. The wells were then emptied and washed with PBS, and 11 µg/mL of resazurin was added. Plates were subsequently read using a Biotek^®^ SynergyH1 UV-Vis spectrometer after 1.5 h of incubation, with excitation at 554 nm and emission at 593 nm. The previously created HEPES buffer contains 10 mM HEPES, 140 mM NaCl and 2.5 mM CaCl_2_ (pH 7.4).

**Statistical analysis:** Data are presented as mean ± standard deviation (SD) unless otherwise indicated. Statistical analyses were performed using GraphPad Prism version 11 (GraphPad Software, Boston, MA, USA). Comparisons among multiple experimental groups were performed using one-way analysis of variance (ANOVA) followed by Tukey’s multiple-comparison test. Pairwise comparisons were performed using an unpaired two-tailed Student’s t-test where appropriate. IC_50_ values were calculated by nonlinear regression using a four-parameter logistic model. Differences were considered statistically significant at *p* < 0.05.

## 3. Results

### 3.1. Structural and Surface Characterization of Biocomposites

**Figure 1:**
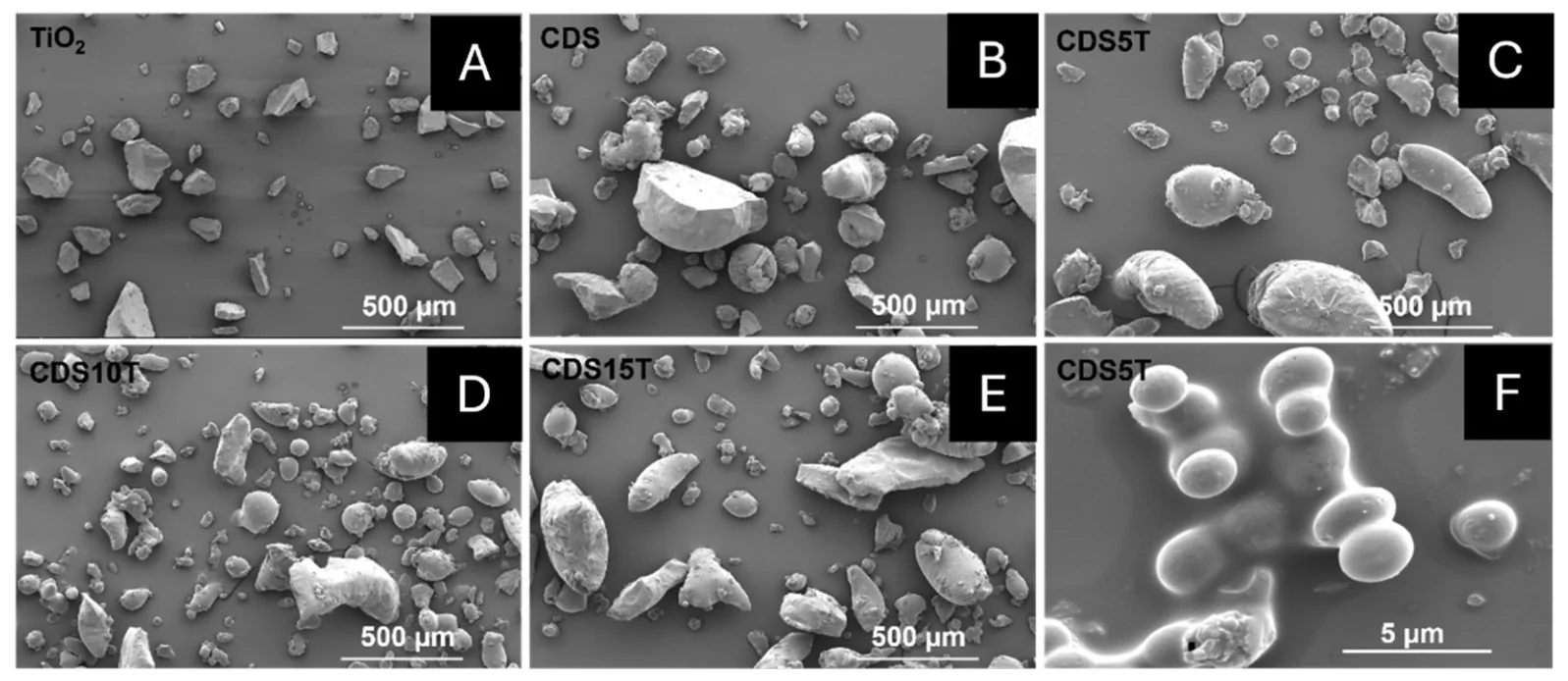
Scanning electron microscopy (SEM) images of N-CD aggregates and TiO_2_ in dissolved form (N-CD_S_5T). Magnification: 100× (**A**–**E**) and 5000× (**F**).

We synthesized nitrogen-doped carbon dot (N-CD) composites made from citric acid and 2,6-diaminopyridine, incorporating different concentrations of TiO_2_ (5, 10, and 15% *w*/*w*). The morphology and size of these structures were characterized by scanning electron microscopy (SEM) (Figure 1). Comparative analysis reveals distinct morphological transitions. Pure TiO_2_ is found in the form of irregular polyhedra. Pure N-CDs exhibit variable morphology, ranging from irregular polyhedral shapes to ovoid or spherical structures. For the composites (N-CDs5T, N-CDs10T and N-CDs15T), the incorporation of TiO_2_ significantly alters the structural organization. Unlike the isolated precursors, the resulting composites lose their initial irregular polyhedral geometry, suggesting hybrid integration or coating of the TiO_2_ particles by the carbon dot matrix. A closer look at composites reveals that they are actually made up of a set of smaller particles when viewed at a zoom level of 150. The size of all composite aggregates is predominantly between 100 and 200 µm (Supplemental Figure S1).

**Figure 2:**
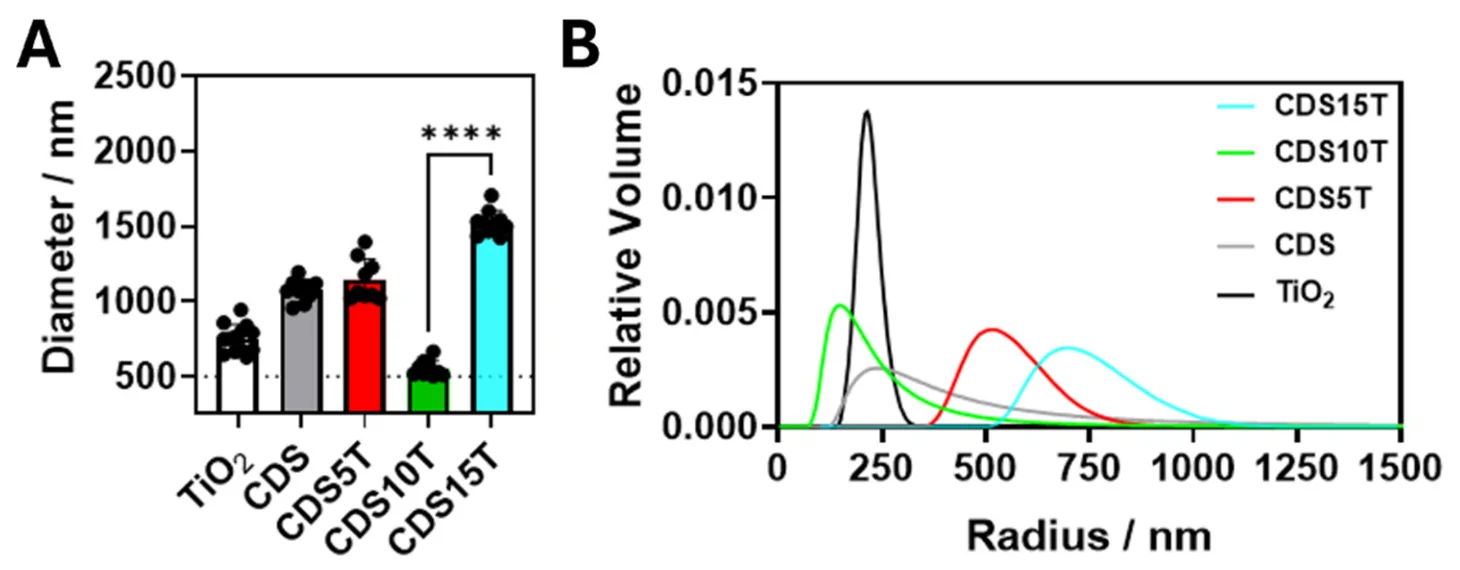
Particle diameter and relative volume distribution using DLS. (**A**), Particle diameter as a function of particles (*n_min_* = 9, *p* = 0.0001). (**B**), Relative volume distribution as a function of radii (examples representing 1 curve). Statistical test: Wilcoxon–Mann–Whitney. *n* = 9 independent replicates.

Observation of the composites in powder form revealed a marked tendency toward aggregation, forming micrometric clusters. Particle size analysis using dynamic light scattering (DLS) further confirmed differences between the biocomposite formulations. Hydrodynamic diameters were measured as 572 ± 93, 1072 ± 75, 1143 ± 140, 554 ± 51, and 1522 ± 83 nm for TiO_2_, N-CDs, N-CDs5T, N-CDs10T, and N-CDs15T particles, respectively (Figure 2). TiO_2_ and N-CDs10T can be classified as nanoparticles, while the others are classified as microparticles. The difference between the N-CD_S_10T and N-CDs15T particle diameter was statistically significant (*p*-value < 0.0001). Examination of the relative volume distributions reveals distinct dispersion profiles depending on the sample composition. The narrowest peaks are observed for pure TiO_2_ and the N-CDs10T biocomposite, indicating a monodisperse character and a homogeneous size distribution within these suspensions. Conversely, the other samples exhibit significantly broader peaks, characteristic of greater polydispersity. This spread of the distribution indicates a wide range of sizes and confirms the presence of aggregates within the liquid medium, particularly for samples deviating from the optimal ratio of 10% (Figure 2). Indeed, the Full Width at Half Maximum (FWHM) of the curves is 1.65, 17.98, 2.81, 1.88 and 2.36° for 62.03, 233.73, 209.33, 147.42, and 259.52 nm for the TiO_2_, N-CDs, N-CDs5T, N-CDs10T, and N-CDs15T, respectively, with a crystallite size of 6.07, 0.45, 4.47, 7.24, and 4.86 nm according to the Debye–Scherrer formula. The results identify N-CDs10T as the most favorable nanocomposite formulation with respect to particle size and dispersion properties.

**Figure 3:**
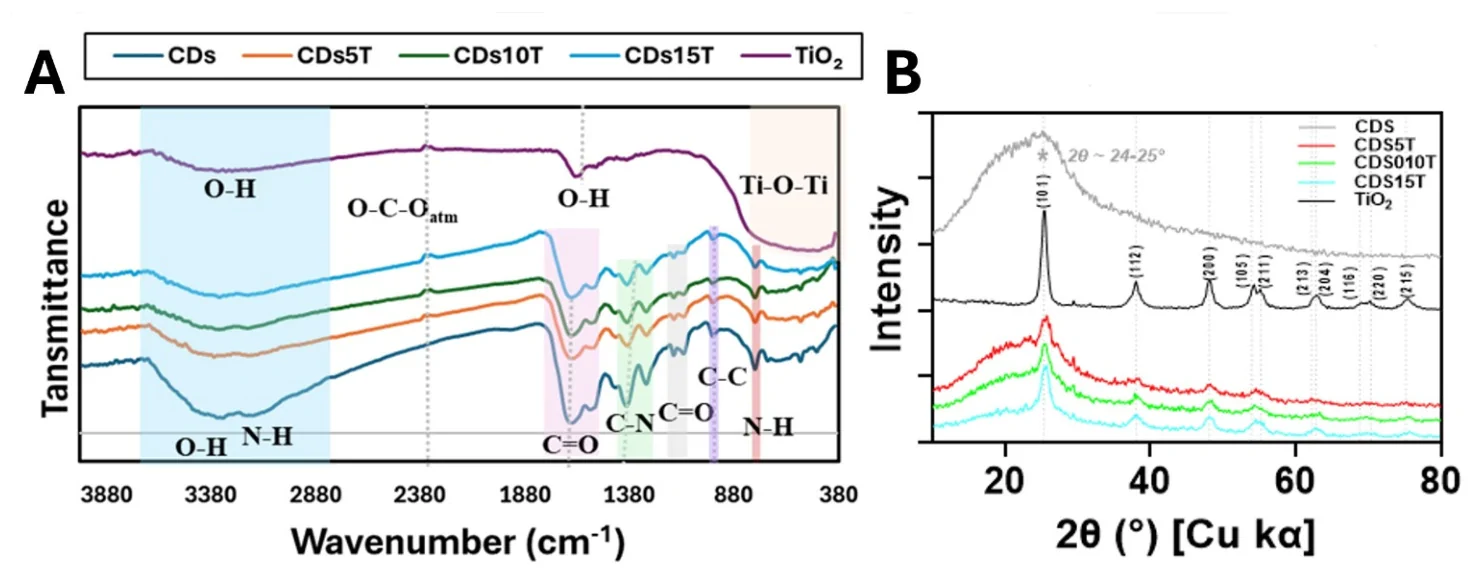
FTIR spectra of synthesized N-CDs (**A**) and XRD pattern of N-CDs and TiO_2_ particles in anatase form (**B**).

Surface functional groups were analyzed using Fourier-transform infrared (FTIR) spectroscopy (Figure 3). The N-CDs exhibited characteristic absorption bands associated with hydroxyl and amine groups in the 2500–3700 cm^−1^ region, indicating a highly hydrophilic surface. A distinct peak near 1660 cm^−1^ corresponded to C=O stretching vibrations, confirming the presence of carboxyl or amide functionalities. Signals observed between 1392 and 1400 cm^−1^ were assigned to C-N stretching, supporting successful nitrogen incorporation within the carbon framework. Additional bands at approximately 1186 cm^−1^ and 1000 cm^−1^ were attributed to C-O and C-C vibrations, respectively. Incorporation of TiO_2_ into the carbon dot matrix resulted in a gradual decrease in the intensity of O-H and N-H stretching bands [7,8], consistent with partial surface interactions between TiO_2_ and N-CD functional groups. Moreover, the appearance of characteristic Ti–O vibration bands confirmed the successful hybridization of the metal oxide within the carbon framework [18,19,20]. X-ray diffraction (XRD) analysis further confirmed the structural features of the composites. N-CDs exhibited a broad diffraction peak around 2θ ≈ 24–25°, characteristic of turbostratic carbon structures with partially graphitic ordering. In contrast, TiO_2_ nanoparticles displayed multiple sharp diffraction peaks corresponding to the anatase phase. In the hybrid composites, increasing TiO_2_ content led to a progressive enhancement of crystalline diffraction peaks, indicating the successful integration of anatase TiO_2_ within the carbon matrix [19,20,21,22].

### 3.2. Baseline Cytotoxicity of Composites in A549 and Kelly Cells

**Figure 4:**
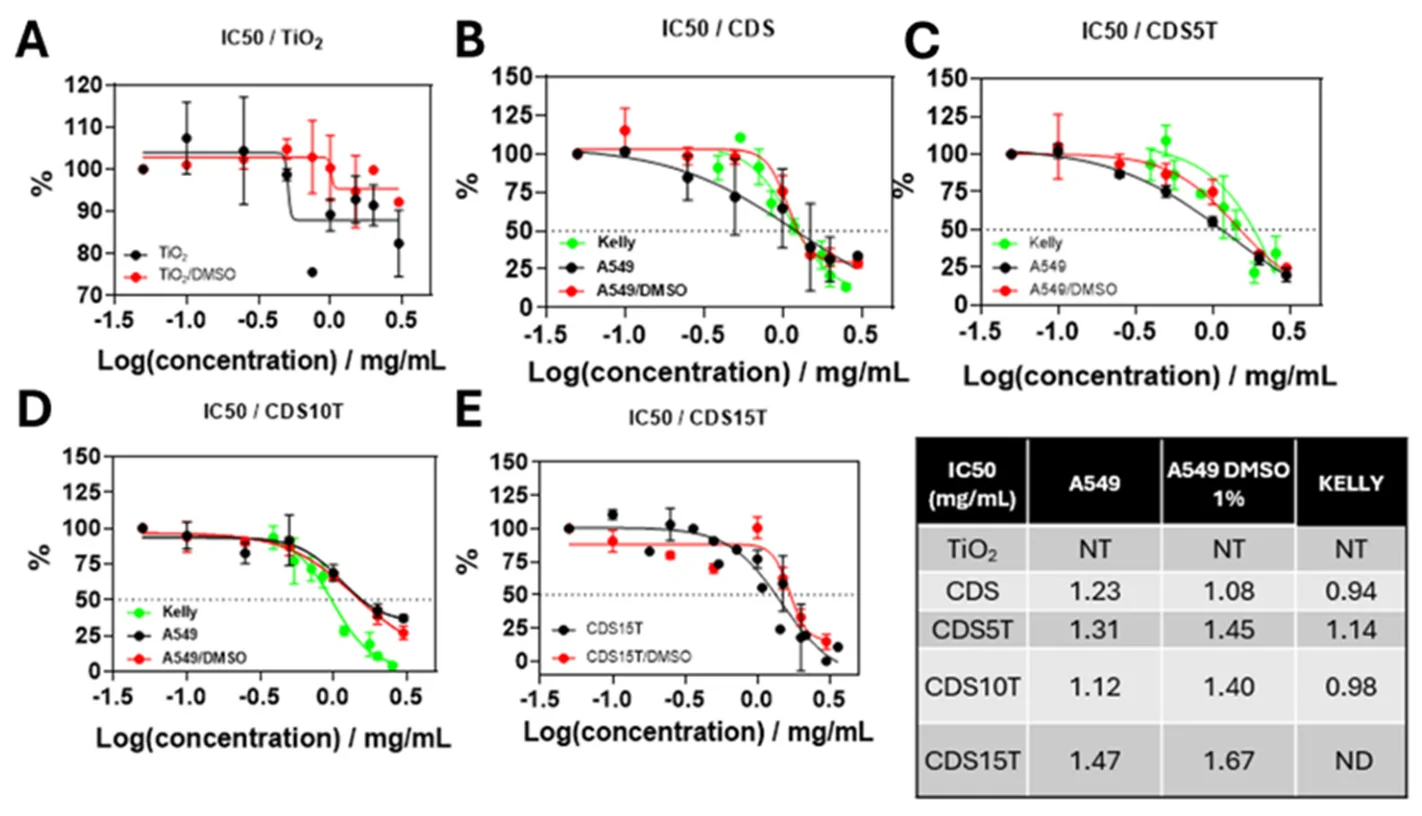
Metabolic activity in A549 and Kelly cells treated with varying concentrations of (**A**) TiO_2_, (**B**) CD_S_, (**C**) CD_S_5T, (**D**) CD_S_10T, and (**E**) CD_S_15T. The table summarizes the IC_50_ values obtained in mg/mL at 24 h with and without DMSO (NT, non-toxic; ND, not determined). The IC_50_ value was calculated for a range of concentrations from 3 to 0.05 mg/mL with n = 3.

The intrinsic cytotoxicity of the synthesized particles was evaluated in A549 lung cancer and Kelly neuroblastoma cells using resazurin-based metabolic activity assays. In the absence of irradiation, all composites displayed relatively low cytotoxicity with IC_50_ values ranging from 0.94 ± 0.15 mg/mL to 1.47 ± 0.24 mg/mL (Figure 4). No significant differences were observed between the various particle formulations, indicating comparable baseline biocompatibility. Importantly, TiO_2_ nanoparticles alone did not induce detectable toxicity within the tested concentration range (0.05–3 mg/mL), confirming that the inorganic component itself does not contribute substantially to dark toxicity. The presence of 1% DMSO used as a solvent control also did not significantly affect cell viability. These observations suggest that the composites possess a favorable safety profile under non-irradiated conditions and may therefore provide a broad therapeutic window for photoactivated applications.

### 3.3. Effects of Composites on Cell Proliferation and Motility

**Figure 5:**
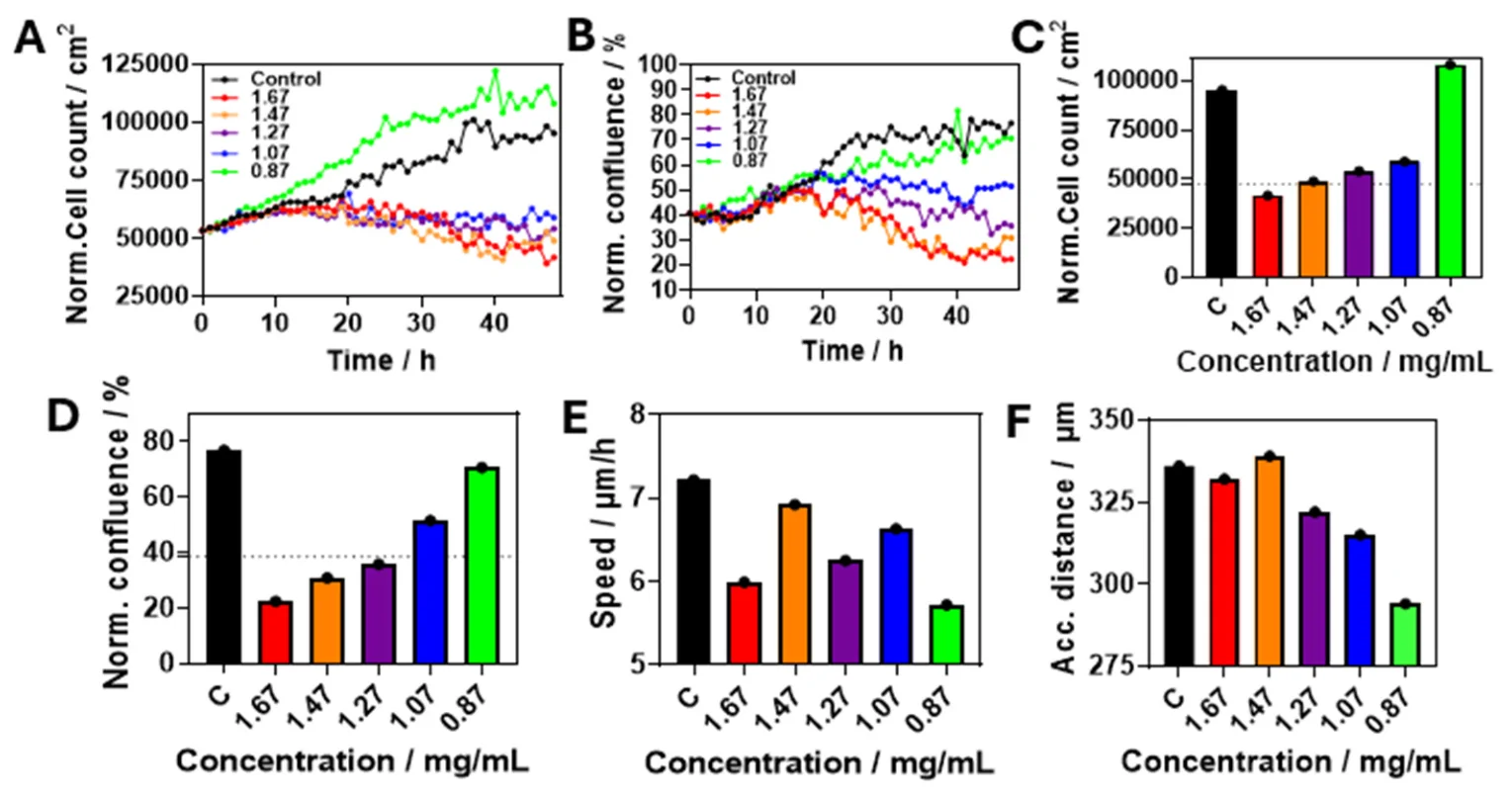
N-CD_S_15T-dependent changes in mobility and confluence of A549 cells. The results were obtained for concentrations of 1.67, 1.47, 1.27, 1.07, and 0.87 mg/mL with 100,000 A549 cells at the start over a period of 48 h. (**A**), Cells counted and normalized per cm^2^ as a function of time (h), (**B**), percentage of confluence normalized as a function of time (h), (**C**), cells counted and normalized per cm^2^ as a function of concentration (mg/mL), (**D**), percentage of confluence normalized as a function of concentration (mg/mL), (**E**), velocity in µm/h as a function of concentration (mg/mL), and (**F**), accumulated distance in µm as a function of concentration (mg/mL).

To further assess the toxicity of our composites, A549 cell proliferation and migration were monitored in real time using holographic live-cell imaging. Cells treated with increasing concentrations of N-CDs15T displayed a clear concentration-dependent reduction in proliferation over a 48 h observation period (Figure 5). Quantitative analyses (Figure 5A–D) allowed us to determine IC_50_ values of 1.47 mg/mL based on the number of cells and of 1.27 mg/mL in terms of confluence. A complementary experiment, conducted under identical conditions, ruled out the hypothesis of a predominant cytostatic effect at low doses (Figure S2). This suggests that the reduction in cell growth at the highest concentrations is rather caused by a cytotoxic effect than via a simple blockage of the cell cycle.

In addition to proliferation effects, treatment with N-CD_S_15T also reduced cell motility, as reflected by decreased migration velocity and accumulated displacement distance. These findings indicate that the composites influence both proliferative and migratory cellular behaviors.

### 3.4. Optical Properties of N-CD/TiO_2_ Composites

**Figure 6:**
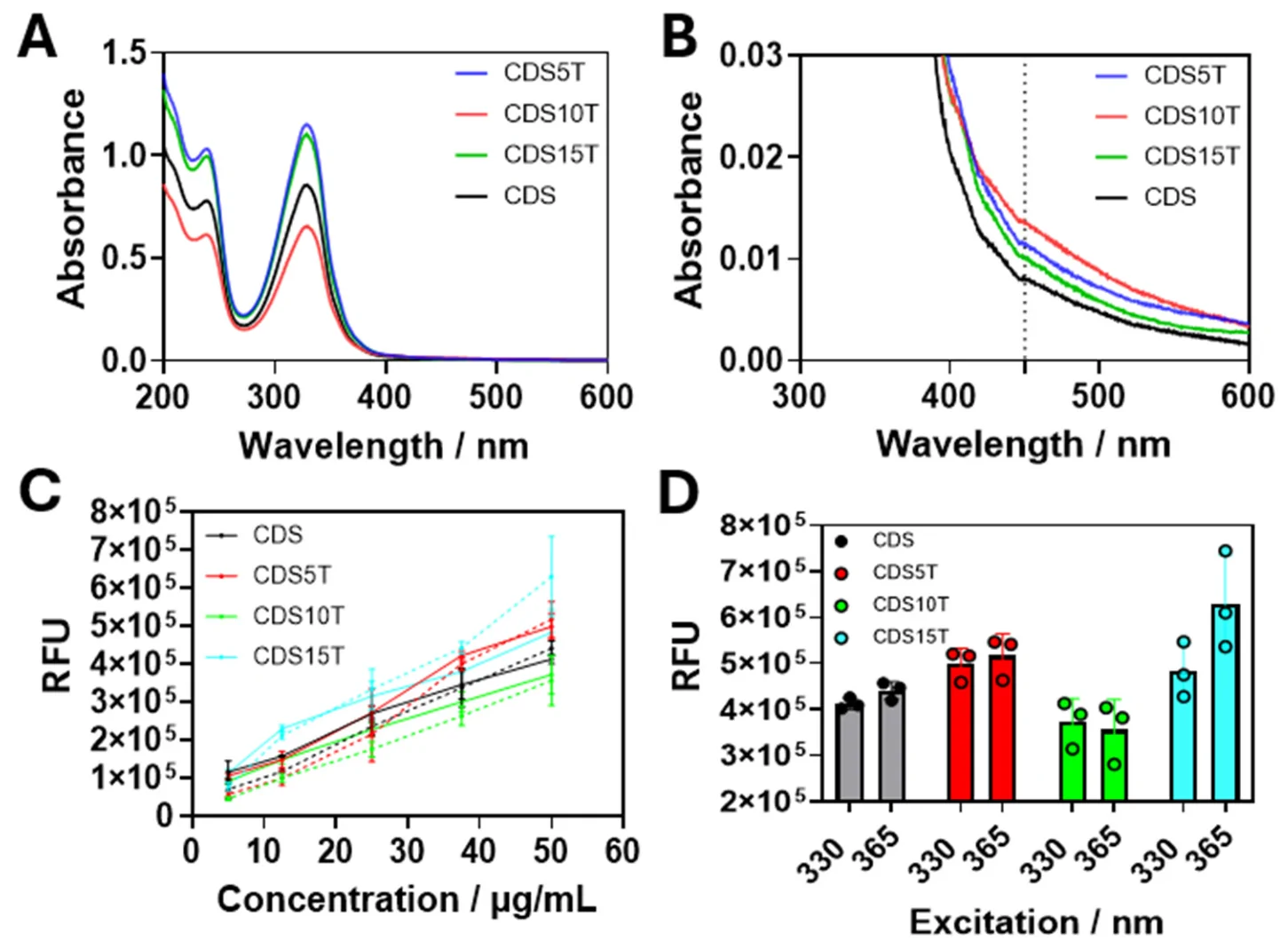
UV–visible spectra of particles in absorbance (qualitative approach) and fluorescence intensity (quantitative approach). (**A**), Absorbance spectra of N-CDs and (**B**), zoom on the range between 300 and 600 nm. (**C**), Fluorescence intensity measured with excitations at 330 nm and 365 nm and emissions at 360 nm and 395 nm as a function of concentration from 5 to 50 µg/mL. (**D**), Data from Figure (**C**) for concentrations of 50 µg/mL are repeated. All data are derived from n = 3 independent experiments.

To determine the optimal irradiation wavelength for singlet oxygen production, the UV–visible absorbance spectra of the composites were analyzed. For all particles, the absorption maxima were located in the range of 238 nm to 330 nm. At 450 nm, a marked hyperchromic effect was observed specifically for the N-CDs10T sample (Figure 6A). Interestingly, although the absorbance intensity between 238 and 330 nm was lower than that of the other samples, N-CDs10T exhibited the most intense maximum at 450 nm, suggesting enhanced electronic interactions between N-CDs and TiO_2_ particles (Figure 6B). In terms of fluorescence intensity, N-CDs15T and N-CDs5T displayed the highest intensity measurements (excitation: 365 nm; emission: 395 nm), with averages of 6.31 × 10^5^, 3.51 × 10^5^, 5.11 × 10^5^, and 4.41 × 10^5^ for N-CDs15T, N-CDs10T, N-CDs5T, and N-CDs, respectively (Figure 6C,D).

### 3.5. Irradiation-Dependent ROS Generation

**Figure 7:**
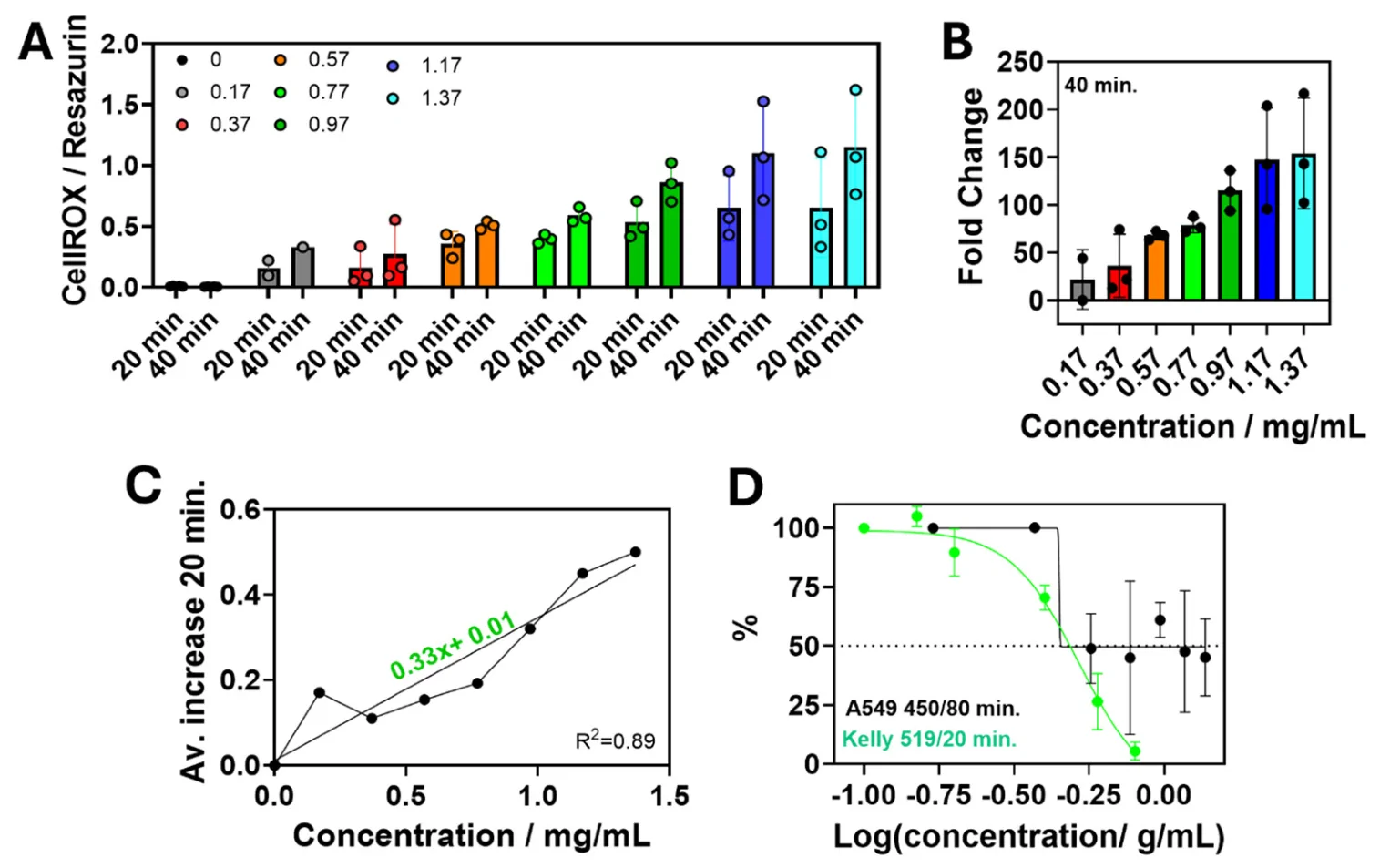
CellROX^TM^ ratio and viability intensity (resazurin) as a function of N-CDs10T concentration and irradiation time. (**A**), Ratio of fluorescence intensity to viability intensity as a function of N-CDs10T concentration and irradiation time. (**B**), Fold change at 40 min of irradiation compared to cells irradiated without N-CDs. (**C**), The average increase in the ratio over the last 20 min of irradiation as a function of the concentration of N-CDs10T. (**D**), IC50 of N-CDs irradiated for 80 min and non-irradiated N-CDs. All graphs are derived from n = 3 independent experiments.

**Figure 8:**
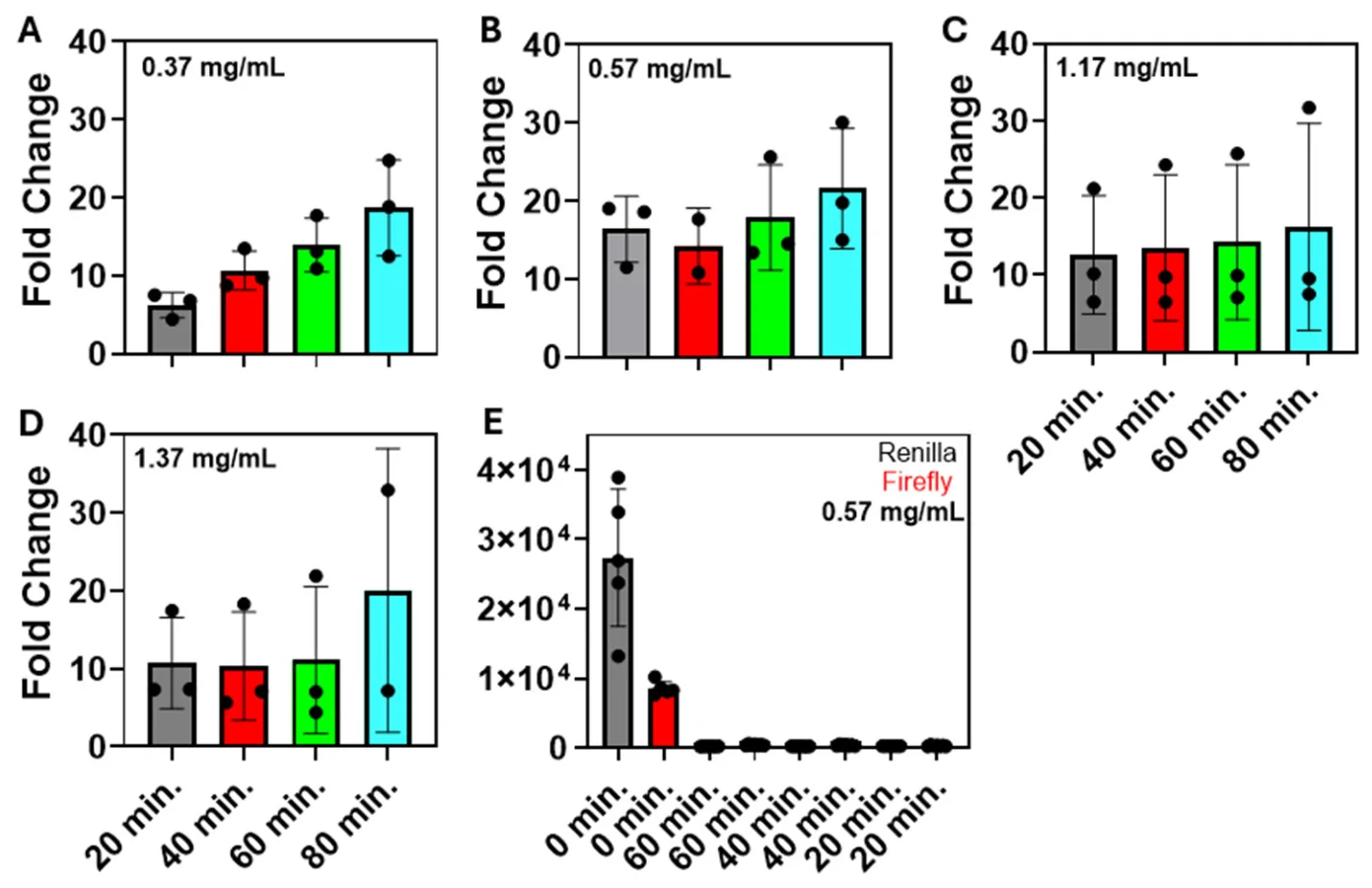
Fold change ratios of H_2_DCF-DA intensity and viability intensity (resazurin) as a function of N-CDs10T concentration (mg/mL) and irradiation time (min). N-CDs10T-dependent changes in antioxidant response element (ARE)-driven luciferase activity. (**A**–**D**), Fold change ratios of H_2_DCF-DA irradiated at 20, 40, 60, 80 min. (**E**), Luciferase activity irradiated at 0, 20, 40, 60 min. All graphs are derived from n = 3 independent experiments with A549 cells. Fold change ratios are based on cells irradiated and not treated with N-CDs10T.

To determine whether the observed cell death is correlated with ROS production, two complementary fluorescent probes were used. Although the CellROX™ Green probe targets nuclear DNA and is known for its photostability (Figure 7), the H_2_DCF-DA probe is localized in the cytosol and is particularly sensitive to light (Figure 8). We observed that the ratio between ROS fluorescence intensity and viability increases proportionally to N-CDs10T concentration and irradiation time (Figure 7A). After 40 min of irradiation, the ratio is 0.007 ± 0.002 for control cells (without N-CD_S_), while it reaches 1.15 ± 0.43 for cells treated with 1.37 mg/mL of N-CDs10T. Cells exposed to the nanocomposite in the absence of irradiation exhibited ROS levels approximately ten-fold lower than those observed under irradiation conditions, confirming that ROS generation is primarily light-dependent. Regarding kinetics and photodynamic efficiency, we observed that the increase at 20 min of irradiation follows a linear regression (y = 0.33x + 0.01, Figure 7C). At 40 min, the induction factors (fold changes) of ROS fluorescence vary from 0 to 200 depending on the N-CD dose (Figure 7B). After 80 min of irradiation, significant cell detachment was observed. Under these conditions, the IC_50_ dropped to 0.44 mg/mL (Figure 7D). Unlike for A549 cells, 20 min of irradiation was sufficient for Kelly cells (IC_50_ = 0.519 mg/mL).

Although the H_2_DCF-DA probe is recognized for its increased sensitivity, its specificity is less than that of the CellROX™ probe, as it reacts to a broader spectrum of species, including hydrogen peroxide (H_2_O_2_). With the exception of the 0.37 mg/mL concentration, no significant difference was observed between the different irradiation durations (Figure 8). This suggests rapid probe saturation or stabilized cytosolic ROS production kinetics. A difference of a factor of 10 is observed between cells irradiated alone and those irradiated in the presence of N-CDs10T, suggesting an active role of the composites in inducing oxidative stress. Strikingly, fluorescence intensity is 1000 times greater in cells irradiated with N-CDs10T compared to cells containing N-CDs10T kept in the dark (Figures S3 and S4). In a completely independent study, we used luciferase reporter assays regulated by the antioxidant response element (ARE). Exposure to UV radiation in the presence of N-CDs10T led to rapid activation of oxidative stress pathways, with detectable effects as early as 20 min after irradiation, confirming that ROS generation is primarily triggered by photoactivation (Figure S5). Notably, doping with 10% TiO_2_ increased the rate of ROS generation after 20 min of irradiation by 47% for the H_2_DCF-DA probe and by 66% for the CellRox™ probe (Figure S7).

### 3.6. Induction of Apoptosis and Cell-Cycle Arrest

**Figure 9:**
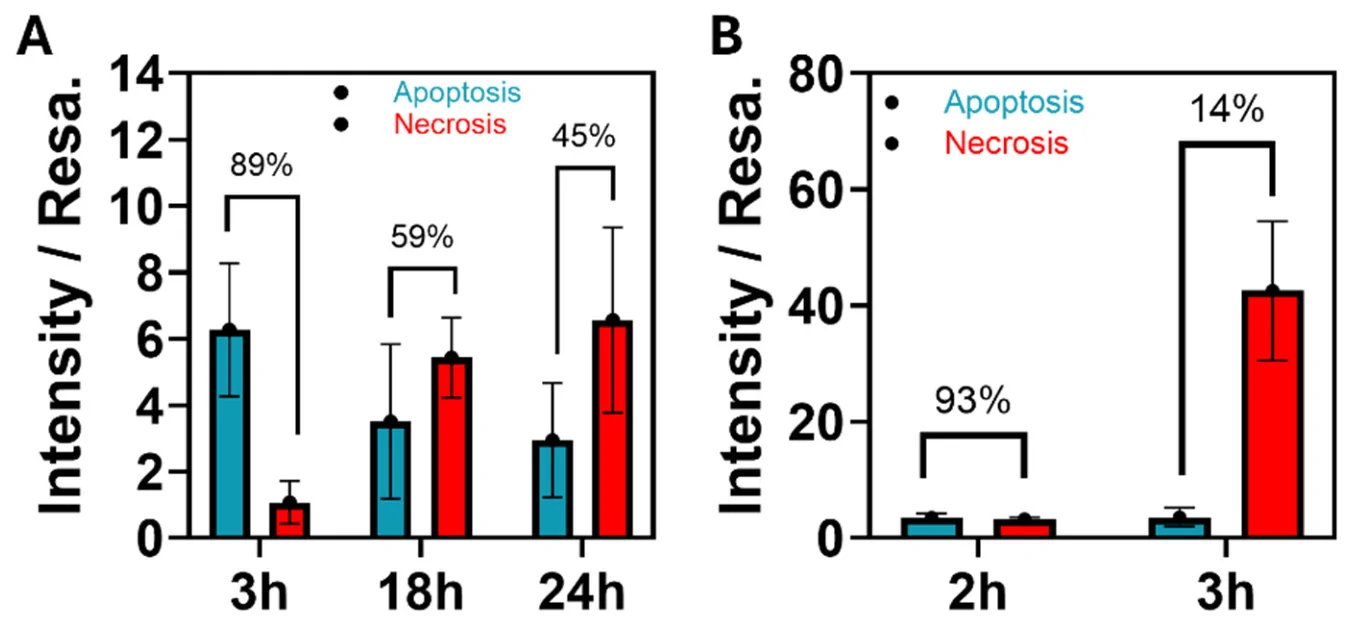
Comparative assessment of the kinetics of induced apoptosis and necrosis in A549 (**A**) and Kelly (**B**) cell lines. All graphs are derived from n = 3 independent experiments (the figure shows the means and standard deviations).

Analysis of N-CDs10T on A549 cells revealed early and progressive cytotoxicity (Figure 9A), with viability dropping from 89% at 3 h to 45% at 24 h (1.5 mg/mL). While early death is driven by structured apoptosis, the later stages show a dramatic shift toward secondary necrosis, likely due to metabolic exhaustion. Surprisingly, it only took 2 h for Kelly cells, with a lower concentration (1 mg/mL), to achieve massive cell necrosis (86%) (Figure 9B).

Flow cytometric analysis of PI-stained cells was performed to evaluate the effect of N-CDs10T (1.5 mg/mL, 24 h) on cell-cycle progression in A549 cells (Figure S6). Treatment with N-CDs10T altered cell-cycle distribution, predominantly inducing an accumulation of cells in the G1 phase (67.3 ± 13.6% in treated cells vs. 60.2% in non-treated controls), reaching up to 78.9% in individual replicates (N3). Concomitantly, a subset of treated cells exhibited a transient retention in the S phase (up to 36.9% in N1 vs. 28.7% in control), reflecting a heterogeneity in the cellular stress response or transient replicative arrest prior to cell death. The proportion of cells in the G2/M phase remained largely comparable to control conditions (6.2 ± 0.6% vs. 5.17%). These results demonstrate that photoactivated N-CDs10T disrupts normal cell-cycle dynamics, primarily by inducing a G1-phase arrest alongside heterogeneous replicative stress across cell populations.

Collectively, these findings indicate that photoactivated N-CDs10T nanocomposites induce oxidative stress-mediated cytotoxicity associated with apoptosis, necrosis, and disruption of cell-cycle progression [23,24].

## 4. Discussion

Nitrogen-doped carbon dots (N-CDs) have emerged as promising photoresponsive nanomaterials because of their tunable optical properties, facile synthesis, excellent aqueous dispersibility, and relatively low intrinsic toxicity. In recent years, substantial efforts have focused on enhancing their photodynamic efficacy through heteroatom doping [25,26,27] and hybridization with semiconductor materials capable of improving charge separation and ROS generation following photoactivation [28,29,30]. Recent reviews have highlighted these approaches as promising strategies for the development of next-generation photosensitizers for photodynamic therapy (PDT) [4,5,6]. In the present study, we systematically investigated how the composition of N-CD/TiO_2_ hybrid composites influences both their physicochemical characteristics and their biological activity. By integrating comprehensive material characterization with complementary biological assays, we demonstrate that subtle differences in hybrid composition translate into marked differences in oxidative stress signaling, cell-cycle regulation, apoptosis, proliferation, and migration following light activation.

### 4.1. Physicochemical Properties of the Hybrid Composites

Among the three formulations investigated, N-CDs10T consistently exhibited the most favorable combination of physicochemical characteristics and biological activity. DLS measurements revealed the smallest hydrodynamic diameter together with the narrowest particle size distribution, suggesting improved colloidal stability compared with the other formulations. Although nanoparticle size can influence interactions with biological systems, hydrodynamic diameter alone cannot predict cellular uptake or intracellular localization. We therefore deliberately avoid concluding that the smaller particle size of N-CDs10T resulted in greater cellular internalization, as uptake was not directly investigated in the present study. At first glance, the micrometric aggregates observed by SEM appear inconsistent with the nanometric particle sizes measured by DLS. However, these techniques characterize different physical states of the material. The SEM images are acquired from dehydrated samples after solvent evaporation, a process that commonly promotes particle aggregation, whereas DLS determines the hydrodynamic diameter of particles dispersed in an aqueous suspension. Similar discrepancies between electron microscopy and DLS have been widely reported for nanomaterials and should therefore be regarded as complementary rather than contradictory observations. Optical characterization likewise contributed to the selection of N-CDs10T for detailed biological analyses. Interestingly, the formulation producing the strongest biological responses was not the one exhibiting the highest fluorescence intensity. This observation is entirely consistent with the photophysics of photosensitizers. Fluorescence emission and ROS generation represent competing de-excitation pathways of the excited singlet state. Efficient photodynamic activity depends primarily on intersystem crossing to long-lived triplet states capable of generating ROS through Type I and/or Type II photochemical mechanisms rather than on maximal fluorescence quantum yield. Consequently, highly fluorescent carbon dots do not necessarily represent the most effective photosensitizers, as has also been reported for several other carbon-dot-based PDT systems [4,5,6].

### 4.2. Photoactivation Drives Oxidative Stress and Downstream Biological Responses

The central finding of the present study is that the biological activity of the N-CD/TiO_2_ hybrids was strongly dependent on light irradiation. Under dark conditions, all formulations displayed relatively modest cytotoxicity, whereas irradiation at 365 nm induced marked ROS production, activation of antioxidant response element (ARE)-dependent signaling, apoptosis-associated cell death, inhibition of proliferation, reduced migration, and alterations in cell-cycle progression. The concordant results obtained using two independent ROS-sensitive fluorescent probes together with activation of the ARE reporter strongly support the conclusion that photoactivation of N-CDs10T induces a robust oxidative stress response. Our findings are in agreement with previous studies demonstrating that nitrogen doping enhances the photodynamic properties of carbon dots by modifying their electronic structure and facilitating photoinduced electron transfer [2,4]. Furthermore, hybridization with TiO_2_ has been proposed to suppress electron–hole recombination, thereby increasing the lifetime of excited charge carriers and enhancing ROS generation [3,5]. Although the present study was not designed to directly investigate charge-transfer mechanisms, the enhanced irradiation-dependent biological activity observed for N-CDs10T is consistent with these proposed photophysical interactions. Both CellROX™ and H_2_DCF-DA detect global oxidative stress rather than individual ROS. Consequently, the present study cannot distinguish between Type I photochemical reactions involving radical formation and Type II reactions leading to singlet oxygen production. Likewise, although UV–visible spectroscopy was used to identify an appropriate excitation wavelength, it does not directly demonstrate singlet oxygen generation. Increasing evidence suggests that carbon-dot-based photosensitizers may operate through mixed Type I and Type II mechanisms, an attractive characteristic for photodynamic therapy because Type I pathways may remain effective under the relatively hypoxic conditions frequently encountered within solid tumors [5]. Direct identification of the predominant ROS using selective fluorescent probes or electron paramagnetic resonance spectroscopy will therefore represent an important objective of future mechanistic investigations. Because ROS fluorescence was normalized to metabolic activity, decreasing cell viability at higher treatment concentrations may partially amplify the calculated ROS/viability ratio. Nevertheless, the irradiation-dependent increase in oxidative stress was consistently observed using both fluorescent probes and was further supported by activation of the ARE reporter assay, indicating that the observed biological effects cannot be explained solely by the normalization procedure.

### 4.3. Differential Sensitivity of Kelly and A549 Cells

An interesting observation was the substantially greater sensitivity of Kelly neuroblastoma cells compared with A549 lung adenocarcinoma cells. Following irradiation, Kelly cells exhibited lower IC_50_ values together with more pronounced apoptosis and necrosis. Although the present study was not designed to elucidate the molecular basis for this difference, several biological explanations appear plausible. Neuroblastoma cells frequently exhibit elevated basal oxidative stress together with a comparatively limited antioxidant reserve, rendering them particularly susceptible to further ROS accumulation. Cell-type-specific differences in glutathione metabolism, mitochondrial function, iron homeostasis, and NRF2-dependent antioxidant responses have all been implicated in determining susceptibility to ROS-mediated therapies. Whether these mechanisms contribute to the enhanced sensitivity of Kelly cells remains to be established but represents an interesting direction for future investigation.

### 4.4. Implications for Photodynamic Therapy

Carbon dots are increasingly recognized as promising next-generation photosensitizers because they combine favorable optical properties with straightforward synthesis, extensive opportunities for chemical modification, and relatively low dark toxicity. Hybridization with semiconductor materials such as TiO_2_ further expands these possibilities by improving photoinduced charge separation and enhancing ROS generation. Recent studies describing carbon dot/TiO_2_ hybrid materials have reported enhanced photochemical performance compared with the individual components [6]. Our work extends these observations by systematically correlating hybrid composition with both physicochemical properties and multiple orthogonal biological responses. Rather than simply demonstrating ROS-mediated phototoxicity, the present study integrates analyses of oxidative stress signaling, cell-cycle progression, apoptosis, proliferation, and migration, thereby providing a more comprehensive biological evaluation of N-CD/TiO_2_ hybrid materials. Importantly, the present work should be regarded as a proof-of-concept study. Irradiation was performed using 365 nm UVA light, which efficiently activates the hybrid materials but exhibits limited tissue penetration and therefore restricts direct clinical translation for deep-seated tumors. Future optimization should therefore focus on shifting excitation toward the visible or near-infrared therapeutic window while maintaining efficient ROS generation. Such developments, combined with improved tumor-targeting strategies and further optimization of hybrid composition, may substantially increase the translational potential of these materials.

### 4.5. Study Limitations

Several limitations should be acknowledged. First, biological evaluation was restricted to two cancer cell lines, and future studies should determine the selectivity of the hybrid materials using non-malignant cells and more physiologically relevant three-dimensional tumor models. Second, although FTIR, XRD, SEM, DLS, and optical spectroscopy collectively support the successful formation of the N-CD/TiO_2_ hybrid composites, complementary structural characterization using techniques such as transmission electron microscopy, X-ray photoelectron spectroscopy, Raman spectroscopy, or thermogravimetric analysis would provide additional insights into the hybrid interface. Third, dedicated TiO_2_ irradiation controls and direct investigation of intracellular uptake would further clarify the relative contribution of each component to the observed biological effects. Finally, the present study quantified global oxidative stress rather than individual ROS and therefore cannot distinguish between Type I and Type II photochemical pathways. These aspects represent important priorities for future investigations.

## 5. Conclusions

In conclusion, this study demonstrates that rational variation in N-CD/TiO_2_ composition substantially influences both the physicochemical characteristics and the photodynamic biological activity of the resulting hybrid materials. Among the formulations investigated, N-CDs10T combined favorable colloidal properties with pronounced irradiation-dependent ROS generation and cytotoxicity while maintaining relatively low dark toxicity. By systematically correlating hybrid composition with oxidative stress signaling, apoptosis, cell-cycle progression, proliferation, and migration, our work extends previous studies beyond simple phototoxicity assays and provides a comprehensive biological evaluation of N-CD/TiO_2_ hybrid materials. Collectively, these findings establish a proof of concept for the rational optimization of carbon dot/TiO_2_ hybrid systems as photoresponsive materials for future photodynamic applications.

## Data Availability

All data are made available within the manuscript and supplemental information.

## Supplementary Materials

The following supporting information can be downloaded at https://www.mdpi.com/article/10.3390/biom16091229/s1, Figure S1: The relative frequency in % of composite size (over length) in µM; Figure S2: Changes in the mobility of A549 cells under the effect of N-CDs15T; Figure S3: Change in the firefly/*Renilla* ratio as a function of irradiation time; Figure S4: Ratios of H_2_DCF-DA intensity and viability intensity (resazurin) as a function of N-CD_S_10T concentration and irradiation time; Figure S5: Ratios of firefly/*Renilla* intensity as a function of N-CD_S_10T concentration (mg/mL) and dual luciferase reporter gene assays (fold change) with A549 cells treated with 0.57, 0.87, 1.17, 1.47 mg/mL N-CDs10T. Figure S6. Cytometry results. Figure S7. TiO_2_-dependent ROS generation after 20 min of irradiation.

## Abbreviations

The following abbreviations are used in this manuscript:

CDs	Carbon Dots
N-CDs	Nitrogen-doped Carbon Dots
PDT	Photodynamic Therapy
ROS	Reactive Oxygen Species
