# Supplementary material for: Nitrogen-Doped Carbon Dot/TiO2 Hybrid Composites Induce Light-Dependent ROS-Mediated Cytotoxicity in Cancer Cells
Source: Biomolecules. 2026 Aug 24;16(9):1229. doi: 10.3390/biom16091229 (PMC13604842; doi:10.3390/biom16091229)
Supplement: Supplementary file 1 [file biomolecules-16-01229-s001.zip › biomolecules-4429986-supplementary.pdf]

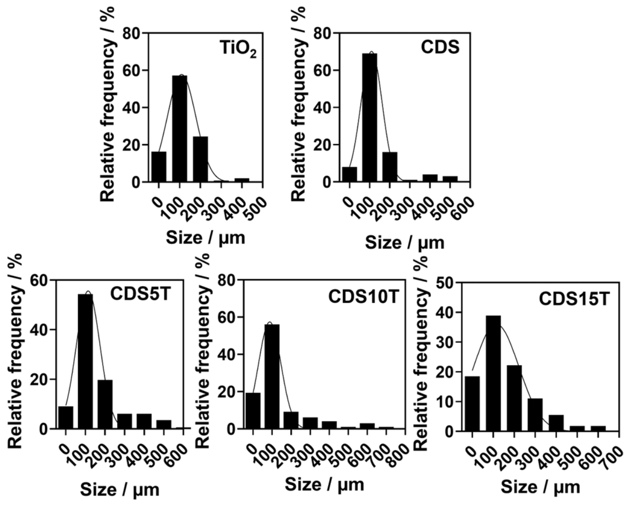

Figure S1. The relative frequency in % of composite size (over length) in µM.

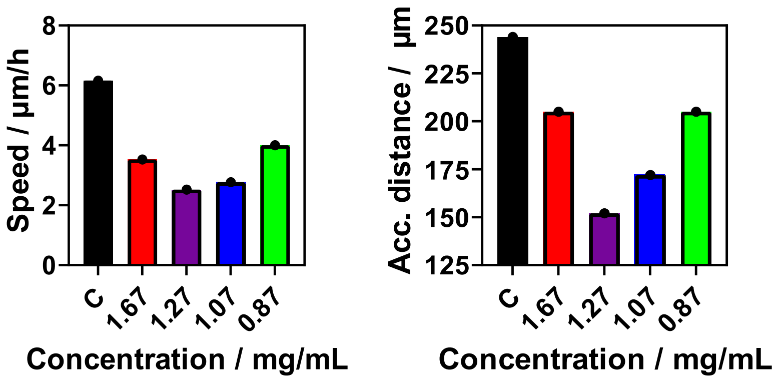

Figure S2. Changes in the mobility of A549 cells under the effect of N-CDs15T.

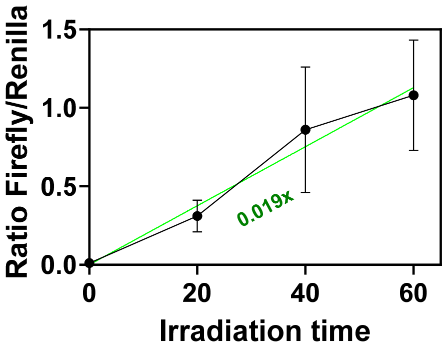

Figure S3. Change in the firefly/*Renilla* ratio as a function of irradiation time.

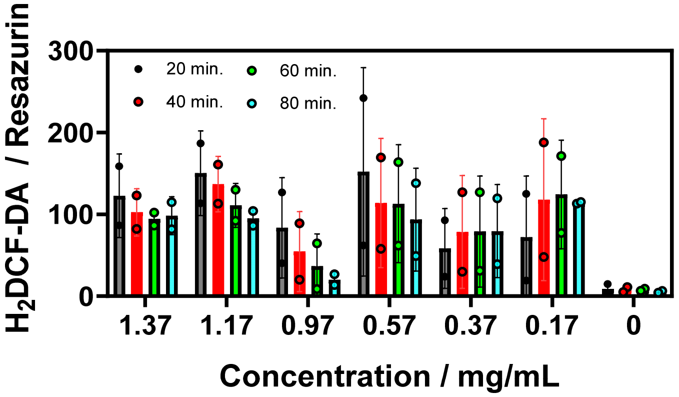

Figure S4. Ratios of H_2DCF-DA intensity and viability intensity (resazurin) as a function of N-CDs10T concentration and irradiation time

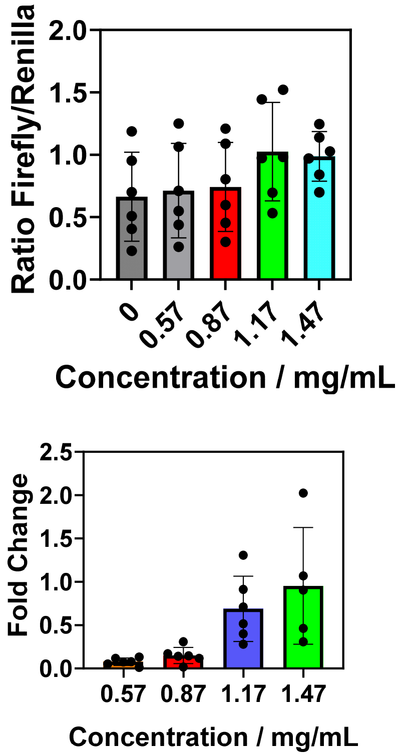

Figure S5. Ratios of firefly/*Renilla* intensity as a function of N-CDs10T concentration (mg/mL) and dual luciferase reporter gene assays (fold change) with A549 cells treated with 0.57–0.87–1.17–1.47 mg/mL N-CDs10T.

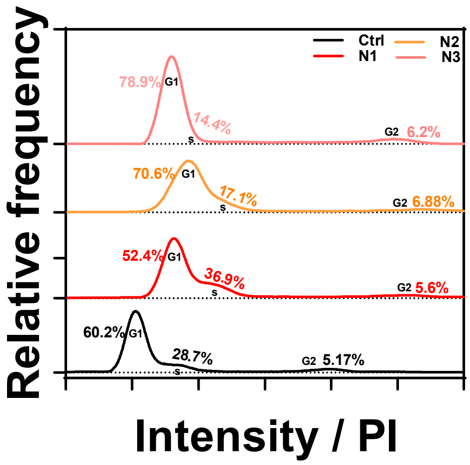

Figure S6. Cytometry results.

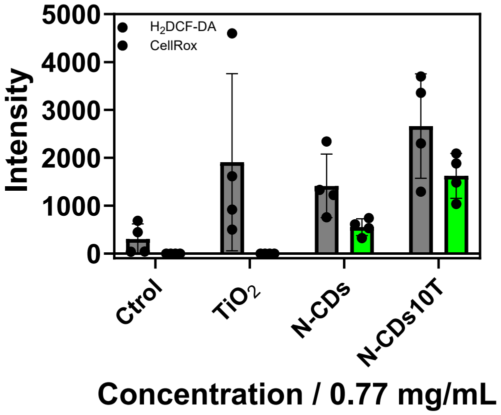

Figure S7. TiO_2-dependent ROS generation after 20 min of irradiation.
